# Editorial: Mental Health and Well-Being Among African Children: Implications of Western Approaches to Counseling and Treatment

**DOI:** 10.3389/fsoc.2021.727587

**Published:** 2021-07-09

**Authors:** Lynne Sanford Koester, Waganesh A. Zeleke

**Affiliations:** ^1^ Department of Psychology, University of Montana, Missoula, MT, United States; ^2^ Department of Counseling, Psychology and Special Education, Duquesne University, Pittsburgh, PA, United States

**Keywords:** African childhood, mental health, counseling African children, autism, adverse experiences, epilepsy, counselor training

For this Research Topic, we invited scholars to submit manuscripts to expand our understanding of the mental health needs and provision of care for children in Africa. The purpose was to enhance our knowledge of the culturally unique experiences of children growing up in African nations, and of the importance of adapting psycho-therapeutic interventions in ways that respect and build upon their inherent strengths.

Africa contains immensely diverse cultures, languages, religions, and resources. The distribution of wealth is highly uneven, resulting in many areas of poverty and others of abundance. From one country to the next, this disparity affects educational opportunities and access to nutritional, sanitation, and health supports necessary for optimal development. In nations struggling to provide the very basics for their citizens, children needing more specialized assistance may be neglected or considered lower priority. Mental health concerns are often addressed primarily through spiritual practices and beliefs. Articles presented here pertain primarily to Ethiopia, Uganda, and Western Africa, but address issues applicable to many other regions of the continent. The mental health needs represented include epilepsy, autism spectrum disorders, trauma and adverse experiences; some focus on counseling techniques and awareness, while others emphasize training or culturally-responsive practices.

Despite great advances in medical care in the developing world, it is still often the case that mental health is not included in these services. The emphasis continues to be on physical health rather than a more holistic approach; funding for research and for training mental health workers reflects this reality in many parts of Africa.

As Sarkar et al. assert, integrating psychological care into the public health system is a global priority inherent in the Sustainable Development Goals. In writing about children with epilepsy in Uganda, these authors explore the perspectives of caregivers in an outpatient mental health clinic, noting that within households there may be differences in the kind of care sought for family members (traditional help, no care at all, or biomedical care). Differences may be related to perceptions of the cause of illness, such as bewitchment or retribution for parental transgressions, in which cases traditional healing is most likely the first form of care sought. Children who show improvements with psychotropic medication are often able to continue this approach, but shortages of free medication may lead to a return to alternative and more traditional therapies.

Clay et al. review the literature regarding interventions with West African children who have endured adverse childhood experiences, finding little evidence that these therapies were designed specifically to meet *children’s* developmental needs. These authors identify four relevant themes: Western, Spiritual, Expressive Arts, and Cultural Approaches. They note that West African children may be exposed to adverse experiences such as terrorism, abuse, and violence due to political conflict, all of which contribute to an increasingly urgent need for mental health interventions. Nevertheless, there is a dearth of information about program availability and efficacy.

Westernized mental health diagnostic processes and evidence-based treatments are limited in many developing countries, as reported by Hughes et al. In Ethiopia, for example, there is often a lack of health care services in general, and therefore an inadequate foundation on which to build mental health practices. Despite a documented need, there are few professional development programs for training mental health workers. Many non-Western cultures adhere to traditional religious views to interpret mental illness; symptoms may be attributed to supernatural causes or spiritual crises, rather than to biopsychosocial influences. As a result, individuals seeking mental health counseling in Ethiopia and many African countries may be limited to family, friends or local healers. Even if families desire to seek Westernized services for their child, there may be barriers such as socioeconomic and cultural factors, negative attitudes (stigma) toward mental illness, or fear of new and unfamiliar practices. Cultural beliefs, traditions, and taboos are passed down through generations. If we are to achieve the goal of improving mental health care access for Africa’s children, a full appreciation of the context and larger cultural considerations is essential.

Wondie and Tadele continue this theme by exploring the extent to which the education system in one Ethiopian town is responsive to the psychosocial and mental health needs of primary school children. In general, teachers’ awareness of their students’ mental health needs tends to be low, and few psychosocial or mental health resources are available. The schools typically do not offer sufficient mental health training to teachers to help them identify problems and make referrals. These authors report that the Ethiopian education system in general is not sufficiently responsive to the psychosocial and mental health needs of primary school students. As in other African nations, there is a critical need for investing in childhood mental health services and appropriate training of relevant professionals.

Zeleke et al. examine the effect of professional development training on educators’ and practitioners’ knowledge of autism spectrum disorders. Helping professionals participated in culturally-responsive and evidence-based training, showing significant improvement in knowledge about the symptoms, nature, and characteristics of ASDs and appropriate interventions. The authors conclude with recommendations for addressing cultural factors impacting the diagnosis and treatment of childhood ASDs in Africa.

According to Meshesha and Johnson, Ethiopia’s Federal Ministry of Health is considering implementing a national mental health strategy guided by the World Health Organization’s pyramid model. This would be a remarkable move toward adopting contemporary approaches to mental health services, but in reality Ethiopia and many African countries struggle with a limited number of well-trained mental health professionals. The authors identify challenges Ethiopia might face in implementing this model, and suggest ways of addressing the critical need for more trained mental health professionals to provide culturally-responsive approaches with children and adolescents.

These articles expand our understanding of mental health issues relevant to African children today, such as the need for better training of personnel working with these children, unique strengths in African families that might lead to more effective interventions, and the importance of building upon these in therapeutic settings.

